# Impact of ultrasonic treatment process on pour point of vegetable oils based liquid insulation

**DOI:** 10.1016/j.ultsonch.2020.105380

**Published:** 2020-10-28

**Authors:** Bakrutheen Moosasait, Willjuice Iruthayarajan Maria Siluvairaj

**Affiliations:** Department of Electrical and Electronics Engineering, National Engineering College, Kovilpatti, Tuticorin, Tamil Nadu, India

**Keywords:** Pour point, Vegetable oil, Liquid insulation, Power transformer, Ultrasonic treatment

## Abstract

This study presents an application of ultrasonic technology in the high voltage liquid insulation domain towards the reduction of pour point of vegetable oil samples for the utilization of vegetable oils as liquid insulation in cold climate areas on power transformers. Pour point reduction has been achieved by processing the vegetable oil samples by using ultrasonic treatment process with 100 W and 30 kHz ultrasonic waves for various exposure times of 15, 30, 45 and 60 min. Edible vegetable oils such as sunflower oil, palm oil, sesame oil and non edible vegetable oils such as honge oil, neem oil and punna oil are considered as two categories of vegetable oils for this experimental investigation. Ultrasonic treatment process results in the reduction of pour point of vegetable oils to meet out the standard value of pour point for liquid insulation as per IEEE Standard C57.147, 2018. A significant reduction in pour point temperature of vegetable oil samples have been obtained with an increased exposure time. The obtained variations in pour point after exposure with ultrasonic waves may be due to the possible changes in crystallization kinetics of fatty acids components of vegetable oil samples due to energy input of ultrasonic waves. The experimental results have given a way towards the positive encouragement and development with ultrasonic treatment for achieving low pour point vegetable oils as liquid insulation in power transformers for applications on cold climatic areas.

## Highlights

• Ultrasonic treatment is used to reduce the pour point of vegetable oil samples.
• The effect of different exposure time is studied on the reduction of pour point.
• Ultrasonic treatment is an effective way of achieving pour point for the cold region.

## 1 Introduction

With the increasing necessity of sustainable solution for different engineering applications, ultrasonic technology has been used by many research and studies as one of the efficient and economic methodology to achieve the desired outcomes. Ultrasonic technology has been utilized in the research fields such as food processing, environmental remediation, and modifications in the physical and chemical properties of gas, vacuum components and fluid characteristics, etc. [1], [2], [3]. As an extension of ultrasonic technology, in the field of high voltage insulation for power transformers, the ultrasonic treatment techniques are employed for reducing the viscosity of vegetable oil based natural esters [4].

Power transformers are one of the most critical electrical power apparatus in the power system setup for uninterrupted power supply to the consumers [5], [6]. For the past few decades, petroleum based mineral oil (namely transformer oil) is used as liquid insulation in the power transformers for the purpose of providing insulation between conductors and as a coolant for transferring heat developed inside transformer winding to the surrounding environment. Mineral oil is preferred as the liquid insulation due to its better electrical, physical and chemical properties [7].

Due course, many researches are carried out for developing the best possible replacement for traditional mineral oil to conquer the facts related to its non-biodegradable nature, for the future availability. Explosive behaviour takes place due to the presence of corrosive sulphur and carcinogenic compounds like poly-chlorinated biphenyl (PCB) and poly-cyclic aromatic hydrocarbons, etc. [5], [8].

From 1980s onwards, many alternate liquid insulation such as silicone oil, less flammable high molecular hydrocarbons, synthetic esters, etc. are proposed as solution to issues related to mineral oil. With the view of developing liquid insulation from natural resources, vegetable oil based liquid insulation is proposed by many researchers since 1900s due to its biodegradable nature, availability and properties. In the due course of time, researchers have recognized that vegetable oil based liquid insulation required some improvements to sort the issues with vegetable oil and to be used as liquid insulation [9], [10].

One of the concerns with vegetable oils is its higher pour point value which will surely influence in the performance of vegetable oils as liquid insulation in cold operating regions [5]. Higher pour point will increase crystallization tendency at low temperature and decrease cold stability. This fact affects the power transformer functioning in low-temperature regions. Hence vegetable oils cannot be implemented directly in those low-temperature regions as liquid insulation in power transformers [11]. For using vegetable oils as liquid insulation, pour point value should be less than −10 °C as per IEEE guide for acceptance of natural esters as liquid insulations, IEEE Std. C57.147, 2018 [12].

Pour point of vegetable oils can be reduced to a specified requirement value by different approaches available in the research techniques. For solving this issue with pour point of the vegetable oils, different approaches are implemented with various processes related to thermal treatment, mechanical treatment, chemical treatment, physical treatment, and acoustical treatment. Some of the important methods used in pour point reductions are transesterification, inclusion of additives and ultrasonic treatment process [13].

Low pour point oil samples can be derived with transesterification processes of free fatty acids when base or acid catalyzed is used with oil samples. This approach is mainly used in the reduction of pour point of heavy crude oil for transportation purpose and developing biodiesel. Addition of complex alcohols produced in the transesterification process in oil samples may increase carbon chains in resulting esters and further reduce the freezing temperature of oil samples. Some of the concerns with the transesterification process is that it takes much time for reaction to get the final products. The requirement of the huge concentration of chemical catalyst is to complete of the reaction, with the involvement of high-cost production, etc. [11].

In petroleum-based products and biodiesel production, the inclusion of additives like pour point depressants such as Poly alkyl methacrylate copolymer, ethylene-vinyl acetate copolymer, Tween-80, dihydroxy fatty acid, acrylated polyester prepolymer, etc. have a fruitful impact on the reduction of pour point. Even 1% of pour point depressant has shown significant results. Inter reactions between hydroxyl functional group of oil samples and pour point depressants might be causing effective reduction in pour point by reducing crystallization and solidification. One concern with pour point depressants is the low solubility in component with high water content. Generally, vegetable oil samples have higher moisture content in their combination along with fatty acids than petroleum based oil samples. Since inclusion of pour point depressants may have a lesser impact on the reduction of pour point of vegetable oil samples [11].

One of the effective approaches implemented in pour point reduction of heavy crude oil samples is an acoustic approach with exposure with ultrasonic waves. Ultrasonic treatment is carried out to different power level and frequency range of ultrasonic wave. This approach is also used in viscosity reduction of heavy oil and vegetable oil based natural esters [4], [13].

Crystallization kinetics is much perceptive towards components in the medium of study. Thermal variations produced due to energy release and modification in molecular structure by simultaneous vibration effects during ultrasonic treatment are the influential factors of solidification process by affecting crystallization kinetics in natural esters. Due to the modification in molecular components and crystallization might be the cause of reduction in pour point from its initial value [13]. Since Ultrasonic treatment approach on pour point reduction is also not involved with the chemical procedure, this method is suitable for producing environmental friendly oil samples. Hence ultrasonic treatment process may be studied for reducing pour point of vegetable oil samples. In this work, the impact of ultrasonic treatment on reduction in pour point of edible and non edible vegetable oils is proposed to study with the experimental procedure.

## 2 Materials and methodology

Vegetable oil is the natural derived products from seed and plants. Vegetable oils are suspended combinations of triglycerides such as saturated fatty acids, mono unsaturated fatty acids and poly unsaturated fatty acids. The triglycerides majorly determine the physiochemical properties of vegetable oil samples [8].

In the proposed experimental work, edible vegetable oils such as sunflower oil (SFO), palm oil (PO) and sesame oil (SO) and non edible vegetable oils such as honge oil (HO), neem oil (NEO) and punna oil (PAO) are considered as two categories of vegetable oils. Vegetable oil samples are purchased from the local manufacturers in refined, bleached and deodorized form. Fatty acid compositions of vegetable oil samples are listed in Table 1.

**Table 1:** Fatty Acid Compositions of Vegetable Oils.

Category	Oil Sample	Fatty Acid Composition		
		Saturated	Unsaturated	
			Mono-	Poly-
Edible Vegetable Oils	SFO	11	30	59
	PO	48	37	9
	SO	14	39	42
Non Edible Vegetable Oils	HO	23	19	58
	NEO	34	14	53
	PAO	14	57	29

Selected base vegetable oil samples are pre-processed to filter out the suspended impurity particles inside the composition of oil samples to meet out with the standard of pure oil samples as specified by CIGRE Work Group’s Study Committee Report 12.17 [14]. Also the excess moisture may be presented in the purchased vegetable oil samples. With the heat treatment for drying process, vegetable oil samples are treated at 80 °C to 100 °C and then oil samples are brought to ambient temperature [15], [16]. The above processed vegetable oil samples are used for further experimentation process.

Ultrasonic treatment process is one of the application areas of ultrasound. Ultrasound is a kind of acoustic signal with a frequency above human hearing range with frequency ranges of 20–60 kHz. Ultrasounds are frequently used in the medical domain and industrial applications [17], [18]. One of the exclusive properties of ultrasound is its capability to change properties of medium and structural composition of any medium by generating vibrations even with low power levels under the exposure of ultrasound for a longer duration [18].

For analyzing the impact of the ultrasonic wave on pour point, selected edible and non edible vegetable oil samples are exposed to ultrasonic waves for exposure durations of 15, 30, 45, and 60 min. Processed vegetable oil samples are brought back to normal condition and further used for measurement of pour point with pour point apparatus based ASTM standard of ASTM D97 [19]. Before and after ultrasonic treatment, pour point temperatures of oil samples are measured for finding the impact of ultrasound.

## 3 Experimental procedure

**Fig. 1:**
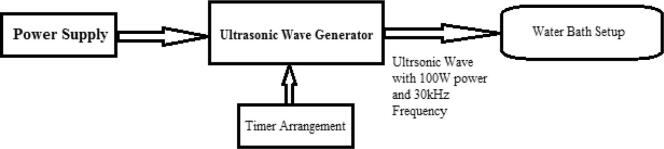
Experimental Setup – Ultrasonic Treatment Process.

In this experimental work, ultrasonic wave generator is used to produce the ultrasonic acoustic wave with 100 W power and 30 kHz frequency. Ultrasonic generator has the setup for holding the oil samples in water bath chamber. Also it has arrangement for varying the operating exposure duration and temperature of water bath arrangement. 50 mL of vegetable oil samples are taken as sample under each category for investigation and the beaker containing sample is placed in the water bath for ultrasonic treatment process. The outline model of experimental setup used for ultrasonic treatment process is shown in Fig. 1.

Low temperature performance is one of the important factors describing the function of liquid insulation in cold climate areas. Pour point of liquid insulation is the lowest temperature at which liquid flows under prescribed conditions. Pour point measurement can be useful in determining the type of liquid insulation for particular equipment in difficult operating conditions such as cold regions [12].

Based on ASTM D97-17b, 2017 [19], pour point is measured with four chamber pour point apparatus. 50 mL of vegetable oil sample is taken in a test tube and placed in copper container which is flooded in cooling medium present inside the pour point apparatus. By reducing the temperature of cooling media through internal arrangements, oil sample in test tube is checked by placing test tube in horizontal manner for solidification. For every 3 °C reduction, pour point temperature is measured at which oil samples get stopped its pouring inside test cell.

## 4 Experimental results and discussion

Experimental investigations are carried out as per methodology and standard procedure for analysing the impact of ultrasonic treatment process on pour point of edible and non edible vegetable oil samples. Pour point of vegetable oils before and after the ultrasonic treatment process and the possible mechanism on the modification of pour point are discussed in this section.

### 4.1 Results of pour point before treatment

Pour point of base vegetable oil samples before undergoing ultrasonic treatment are measured as per standard and the values of pour point are given in Table 2.

**Table 2:** Pour Point of Base Vegetable Oil Samples.

Category	Oil Sample	Pour Point (°C)
Edible Vegetable Oils	SFO	3
	PO	9
	SO	3
Non Edible Vegetable Oils	HO	6
	NEO	3
	PAO	9

From the experimental results of pour point of investigated vegetable oils, it is observed that pour point of vegetable oils are higher than the standard value of −10 °C as per IEEE guide for acceptance of natural esters as liquid insulation for applications in transformers [12]. In the current form of vegetable oils, investigated vegetable oils cannot be used in transformers for cold regions as liquid insulation, because these oil samples may start crystallization process even in positive temperature which will affect cold stability of oil samples [12]. For those cold conditions operations, reduced value of pour point is required for liquid insulation. Hence pour point reduction is carried out with the proposed method of the ultrasonic treatment process.

### 4.2 Results of pour point after treatment

**Fig. 2:**
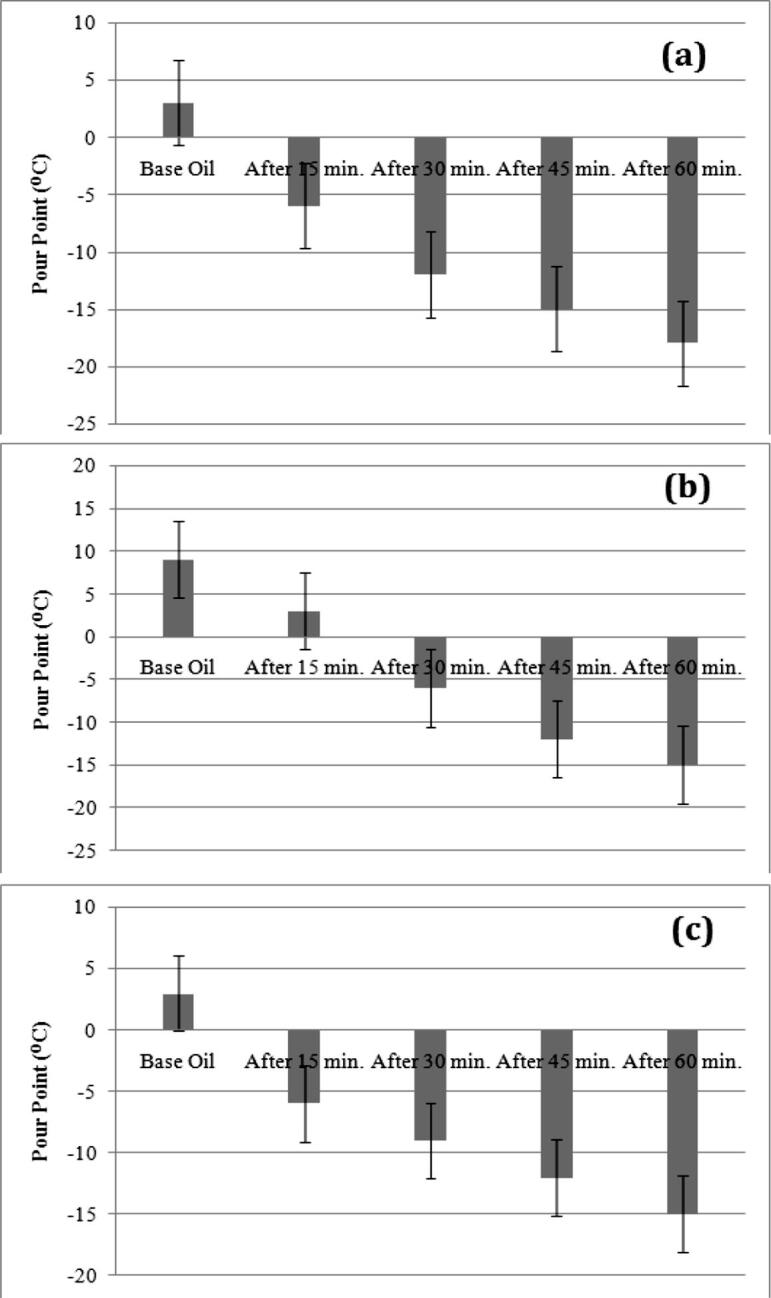
Pour Point of Edible Vegetable Oils – After Ultrasonic Treatment Process (a) For Sunflower Oil, (b) Palm Oil, (c) Sesame Oil.

**Fig. 3:**
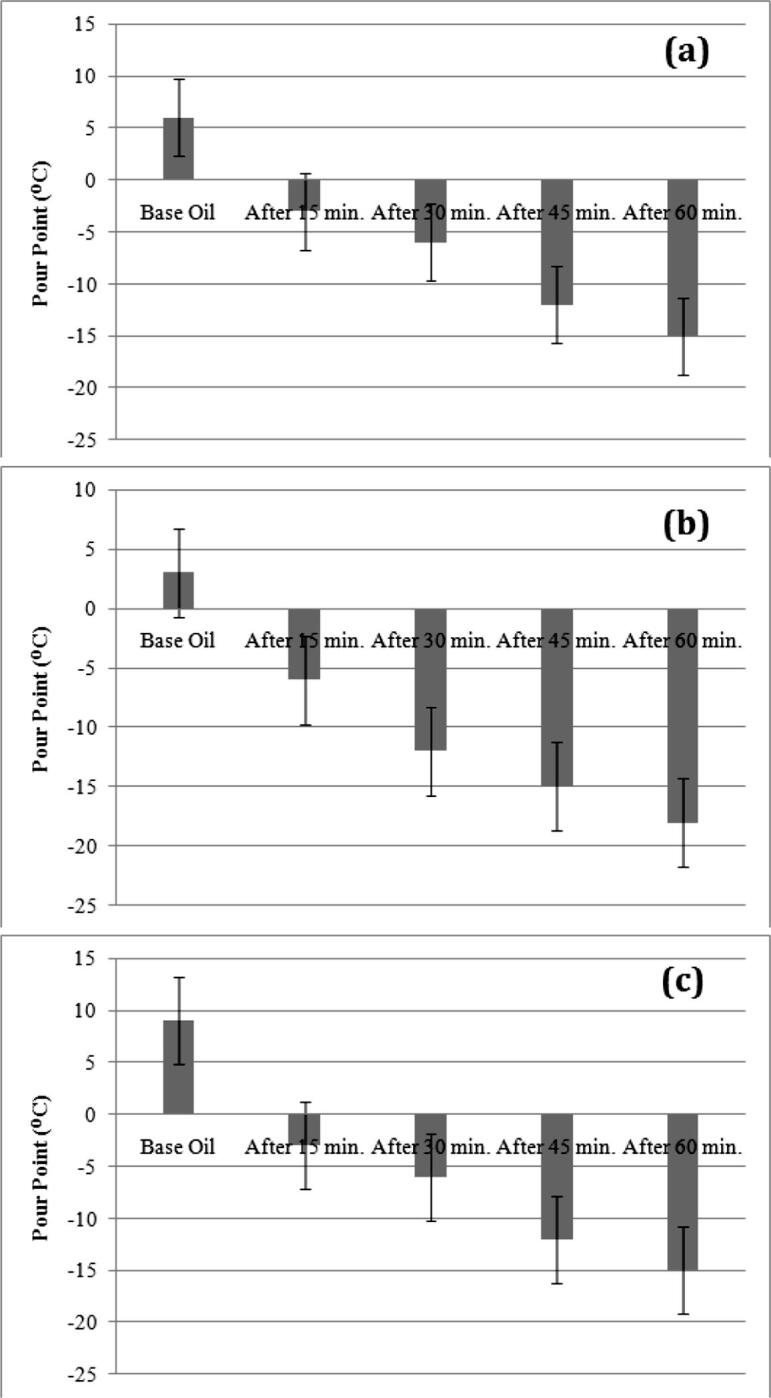
Pour Point of Non Edible Vegetable Oils – After Ultrasonic Treatment Process (a) For Honge Oil, (b) Neem Oil, (c) Punna Oil.

Based on the proposed ultrasonic treatment process, edible and non edible vegetable oil samples are exposed to ultrasonic waves. Pour point of treated oil samples for exposure durations of 15, 30, 45 and 60 min are illustrated in Fig. 2, Fig. 3 respectively for edible vegetable oil samples and non edible vegetable oil samples. The figures are plotted with experimental error bar based on the standard error which will provide the uncertainty data points or statistical data with confidence intervals or the minimum and maximum values in a ranged dataset.

Variations in pour point are analyzed further for studying the effectiveness of the proposed method of pour point reduction in vegetable oils.

From experimental investigations on pour point of edible and non edible vegetable oils before and after exposing with ultrasonic waves, the following observations are inferred.

• All edible and non edible vegetable oil samples have a high value of pour point temperature than the value of pour point specified in the standard.
• Pour point of palm oil is higher than the other two samples under edible vegetable oil category. Similarly, under non edible category, punna oil has a higher pour point temperature.
• After exposure with ultrasonic waves, pour point temperatures of treated vegetable oil samples have shown reduction pattern from its original value.
• Pour point reduction in vegetable oils is relative proportional to exposure time of ultrasonic waves during treatment process. As exposure duration of treatment process increases, pour point temperature is reduced for treated oil samples.
• Differences in pour point temperature from initial value after ultrasonic treatment are listed in Table 3 under different exposure time periods.

**Table 3:** Variations in Pour Point of Vegetable Oil Samples after Ultrasonic Treatment Process.

Oil Samples	Difference in Pour Point from its initial Value (^o^C)			
	Exposure for 15 min	Exposure for 30 min	Exposure for 45 min	Exposure for 60 min
SFO	−9	−15	−18	−21
PO	−6	−15	−21	−24
SO	−9	−12	−15	−18
HO	−9	−12	−18	−21
NEO	−9	−15	−18	−21
PAO	−12	−15	−21	−24
• Vegetable oils are treated for the ultrasonic exposure time with 60 min have a lower value of pour point among investigated vegetable oil samples.
• From pictorial representation, it is observed that variations in pour point reduction are much low in the 60 min exposure compared to the initial decrease of an ultrasonic exposure with the duration of 15 min. This may be an indication of the process moving towards saturation state.
• Reduction in pour point may be occurred due to ultrasonic impact on the molecular composition of vegetable oils and consequence effect on properties of vegetable oil samples.

### 4.3 Possible mechanism for modification in pour point

Low-temperature behaviour of any oil medium is determined with crystallization kinetics of constitute components present in the medium. Since crystallization kinetics of fatty acids influences in pour point value, generally, vegetable oils based liquid insulation have higher value of pour point. Studies on crystalline formation with fatty acids are mostly empirical and such solidification study is not established with traditional approaches. Crystallization kinetics is much sensitive towards temperature variations and components in the investigating medium [13].

**Fig. 4:**
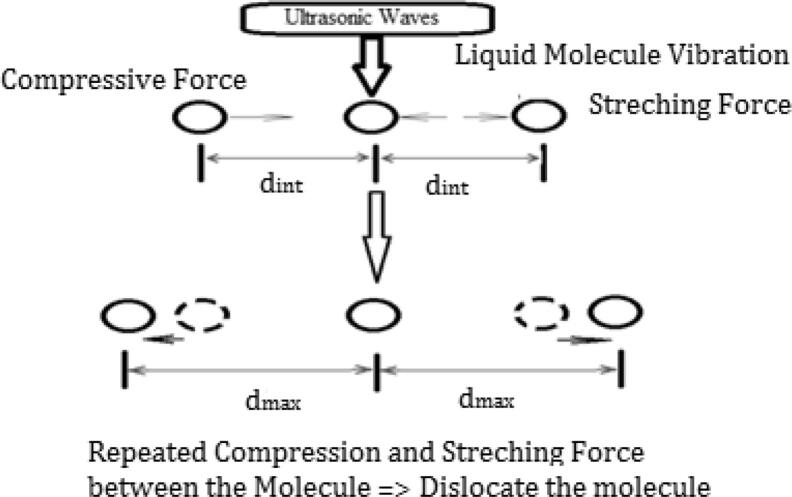
Possible Mechanism of Molecular Dislocation due to Vibrations on Molecule during Treatment with Ultrasonic Waves [20].

The mechanism behind pour point reduction in ultrasonic treated vegetable oil samples is due to different factors related to ultrasonic waves such as ultrasonic power, ultrasonic frequency, and vibration effect [20], [21], [22], [23]. Particularly, vibration produced by ultrasonic waves on the molecular structure of medium is major influential factor in pour point modifications. While ultrasonic wave passes through any medium, it will produce vibration on the molecular structural level. These vibrations will produce repeated compression and stretching forces on molecules present inside the medium and further release more energy which will break the molecular bonding between the structures. The above said process due to vibration results in dislocation of molecule from its original distance between the molecular bonds ‘d_in_’. If the distance of separation goes beyond ‘d_max_’, possibility of holding molecule together is reduced. This kind of changes in molecular structure may be one of the possible reasons for reduction in pour point by modifying the solidification dynamics [20], [21], [22]. The possible process of modification in molecular arrangement is illustrated in Fig. 4.

Vibration level is majorly determined based on power and frequency of ultrasonic waves. Higher frequency has less number of cycles of compression and stretching forces in particular time period. On the basis of effectiveness, lower frequency is capable of producing the molecular structure change than the higher frequency [20], [24], [25], [26].

Modifications in molecular structure by simultaneous vibration effects on vegetable oil samples during ultrasonic treatment process are the possible influential factors of solidification process by affecting crystallization kinetics in vegetable oil samples. Due to the modification in the molecular components and crystallization, pour point temperature of vegetable oil samples may be reduced from its initial value.

Further reduction in the pour point may be due to the changes in phase of materials which may be explained with the concept of energy input. With the energy input (=power*exposure time/volume) for different exposure times of ultrasonic treatment process, there will be a possible conversion of the energy into heat and vibration energy. Further the vibration energy will lead to the formation of cavitations in the molecule by converting the energy as cavitational energy. These energy conversions from ultrasonic energy input into cavitational energy may lead to the alteration on the physical and chemical properties by creating micro streaming [27], [28]. This possible conversion process is completely depends on the exposure time, since the increase in exposure time will again increase the energy input and subsequent possible process.

## 5 Conclusion

Impact of ultrasonic treatment is studied on pour point temperature of the edible and non edible natural esters for analysing its ability to be used in cold climatic regions. For this, the oil samples are exposed with ultrasonic waves of 100 W and 30 kHz for different exposure duration of 15, 30, 45 and 60 min. From the experimentations, it is observed that ultrasonic treatment process on edible and non edible vegetable oil samples esters has a positive impact on achieving lower pour point. Reduction in pour point temperature has a proportional relation with the exposure periods. The possible impact of the ultrasonic wave in crystallization kinetics with molecular modification of molecular composition and impact of exposure time on cavitation process may be the probable reasons behind pour point variation achieved with the ultrasonic treatment process. The ultrasonic treatment has shown its influence on the reduction in pour point of vegetable oil samples, which reduces the pour point temperatures of −15 °C to −18 °C among the various samples for maximum exposure duration. These results are the encouraging factors of utilizing the vegetable oil based insulating liquids in cold temperature region. Further for estimating the consistency of ultrasonic treatment on vegetable oil samples as liquid insulation, impact to be created on other characteristics of vegetable oil samples, influence of power per litre of oil sample, reversible effect on the changes in properties may be analysed with various ultrasonic power level and ultrasonic frequency ranges.

## CRediT authorship contribution statement

**Bakrutheen Moosasait:** Conceptualization, Methodology, Investigation, Writing - original draft. **Willjuice Iruthayarajan Maria Siluvairaj:** Supervision, Writing - review & editing.

## Declaration of Competing Interest

The authors declare that they have no known competing financial interests or personal relationships that could have appeared to influence the work reported in this paper.

## References

[1] Huang D., Men K., Li Dapeng, Wen Tao, Gong Zhong Liang, Sunden Bengt, Zan Wu (2020). Application of ultrasound technology in the drying of food products. Ultrason. Sonochem..

[2] JingWang Z.W., Vieira C.L.Z., Wolfson J.M., Pingtian G., Huang Shaodan (2019). Review on the treatment of organic pollutants in water by ultrasonic technology. Ultrason. Sonochem..

[3] Taheri-Shakib Jaber, Naderi Hassan, Salimidelshad Yaser, Kazemzadeh Ezzatollah, AliShekarifard (2018). Application of ultrasonic as a novel technology for removal of inorganic scales (KCl) in hydrocarbon reservoirs: an experimental approach. Ultrason. Sonochem..

[4] Bakrutheen M., Willjuice Iruthayarajan M., Narayani A. (2018). Influence of ultrasonic waves on viscosity of edible natural esters based liquid insulation. IEEE Trans. Dielectr. Electr. Insul..

[5] Mohan Rao U., Sood Yog Raj, Jarial Raj Kumar (2016). Ester dielectrics: Current perspectives and future challenges. IETE Tech. Rev..

[6] IEEE Standard, C57.91, IEEE guide for loading mineral oil immersed transformer and step voltage regulators, 2011. DOI:10.1109/IEEESTD.2012.6166928.

[7] Fofana I. (2013). 50 Years in the development of insulating liquids. IEEE Electr. Insul. Mag..

[8] Rafiq M., Lv Y.Z., Zhou Y., Ma K.B., Wang W., Li C.R., Wang Q. (2015). Use of vegetable oils as transformer oils – a review. Renew. Sust. Ene. Rev., Elsevier.

[9] Ommen T.V. (2002). Vegetable oils for liquid filled transformers. IEEE Electr. Insul. Mag..

[10] Peppas Georgios D., Charalampakos Vasilios P., Pyrgioti Eleytheria C., Danikas Michael G., Bakandritsos Aristides, Gonos Ioannis F. (2016). Statistical investigation of AC breakdown voltage of nanofluids compared with mineral and natural ester oil, IET Sci. Meas. Tech..

[11] Asadauskas Svajus, Erhan Sevim Z. (1999). Depression of pour points of vegetable oils by blending with diluents for biodegradable lubricants. J. Amer. Oil Chem. Soc..

[12] IEEE Standard. C57.147, IEEE Guide for Acceptance and Maintenance of Natural Ester Insulating Liquid in Transformers, IEEE Power and Energy Society, Newyork, USA, 2018.

[13] Volkova G.I., Anufriev R.V., Yudina N.V. (2016). Effect of ultrasonic treatment on the composition and properties of waxy high-resin oil. Petro. Chem..

[14] CIGRE, Effect of particles on transformer dielectric strength (Working Group 17 of Study Committee 12), 2000.

[15] Rafael David Villarroel Rodríguez, Moisture dynamics in transformers insulated with natural esters, PhD dissertation, Electrical Engineering Department, University Carlos III of Madrid, 2015.

[16] Imran Sutan Chairul, Sharin Ab Ghani, Hidayat Zainuddin, Nur Lidiya Muhammad Ridzuan, Comparative study of moisture removal techniques for mineral based insulation oil, International Multi-Disciplinary Graduate Conference of Terengganu, 2016.

[17] Shol A., Suslick K. (1998). ‘Industrial applications of ultrasound. Ultrasound: Its Chemical, Physical, and Biological Effects.

[18] Cheeke J. (2002). Fundamentals and Applications of Ultrasonic Waves.

[19] ASTM Standard D97-17b, Standard test method for pour point of petroleum products, ASTM International, West Conshohocken, PA, 2017. DOI:10.1520/D0097-17B.

[20] Yusof Siti Mariam, Hussin Nuriziani, Isa Muzamir (2015). Viscosity reduction of palm oil via ultrasonic radiation. Appl. Mech. Mat..

[21] Min Yi, Luo Jian, Liu Chengjun (2019). Viscosity and related structure transformation of fluorine bearing silicate melt under ultrasonic field. Ultrason. Sonochem..

[22] Huang Xintong, Zhou Cuihong, Lanting QuanyuSuo, ShihanWang Zhang (2018). Experimental study on viscosity reduction for residual oil by ultrasonic. Ultrason. Sonochem..

[23] Hamidi H., Rafati R., Junin R., Manan M. (2012). A role of ultrasonic frequency and power on oil mobilization in underground petroleum reservoirs. J. Pet. Explor. Prod. Technol..

[24] Naderi K., Babadagli T. (2010). Influence of intensity and frequency of ultrasonic waves on capillary interaction and oil recovery from different rock types. Ultrason. Sonochem.

[25] Nazari-Mahroo H., Pasandideh K., Navid H.A., Sadighi-Bonabi R. (2018). How important is the liquid bulk viscosity effect on the dynamics of a single cavitation bubble?. Ultrason. Sonochem..

[26] Mohammadian E., Junin R., Rahmani O., Idris A.K. (2012). Effects of sonication radiation on oil recovery by ultrasonic waves stimulated water-flooding. Ultrasonics.

[27] Gregersen Sandra Frydenberg, Hammershoj Rikke, Dalsgaard Marianne, Andersen Trine, Lars Ulf, Wiking (2018). Application of high intensity ultrasound to accelerate crystallisation of anhydrous milk fat and rapeseed oil blends: acceleration of fat crystallization kinetic by ultrasound treatment. Eur. J. Lipid Sci. Technol..

[28] Kasaai Mohamed Reza (2013). Input power –mechanism relationship for ultrasonic irradiation: food and polymer applications. Natural Sci..

